# Recurrent Spontaneous Coronary Artery Dissections in a Male Patient With Fibromuscular Dysplasia

**DOI:** 10.7759/cureus.71783

**Published:** 2024-10-18

**Authors:** Mersal Samimi, Naman Jain, Miro Asadourian, Ashwini P Sadhale, Arang Samim

**Affiliations:** 1 Internal Medicine, University of California San Francisco, Fresno, Fresno, USA; 2 Cardiology, University of California San Francisco, Fresno, Fresno, USA

**Keywords:** acute coronary syndrome (acs) and stemi, coronary catheterization, fibromuscular dysplasia, recurrent spontaneous coronary artery dissection, spontaneous coronary dissection

## Abstract

Spontaneous coronary artery dissection (SCAD) is a rare presentation of acute coronary syndrome characterized by a tearing of the wall of the epicardial coronary artery, leading to myocardial ischemia. SCAD predominantly affects young and middle-aged women, but young men without significant cardiovascular risk factors can also present with the disease. In the setting of fibromuscular dysplasia, men may be at a higher risk for recurrence. We present the case of a young man with a history of fibromuscular dysplasia and prior SCAD in the diagonal branch and right posterolateral artery who presented with exertional chest pain. Further evaluation revealed a recurrent SCAD of the distal right coronary artery despite being on conservative management with aspirin and beta-blockers. There is a need to study the pathophysiology of fibromuscular dysplasia and SCAD in men further and develop medical therapies to treat recurrent SCAD.

## Introduction

Spontaneous coronary artery dissection (SCAD) is an uncommon presentation of acute coronary syndrome (ACS), accounting for about 1-4% of ACS and 35% of myocardial infarction in young women under the age of 50 [1]. SCAD involves a tearing of the inner intimal wall of the coronary artery, which can either create a false lumen or an intramural hematoma, leading to ischemia of the myocardium. This can lead to ACS and myocardial infarctions, usually in younger patients without atherosclerotic disease. 

SCAD predominantly affects young and middle-aged women, but young men can also present with SCAD, especially in those who present with chest pain and no other cardiovascular risk factors. Men represent a minority of SCAD cases, with recent studies estimating about 10% prevalence, which is why SCAD in men has been rarely described in the literature [2]. One study explored the precipitating factors of SCAD between men and women, with intense physical activity and smoking increasing the risk in men compared to pregnancy and fibromuscular dysplasia (FMD) in women [3]. However, FMD can also affect men, weakening arterial walls and worsening dissections and aneurysms. It is crucial to further explore male patients with SCAD and FMD as they can develop severe vascular manifestations and higher rates of recurrence compared to female patients [4]. 

There is a need to evaluate further the risk factors, presentation, management, and complications of SCAD and FMD in the male gender, as sex-based differences can affect long-term outcomes. We will present a case of recurrent SCAD in a young man with a history of FMD despite being on conservative management with aspirin and beta-blockers. 

## Case presentation

**Figure 1:**
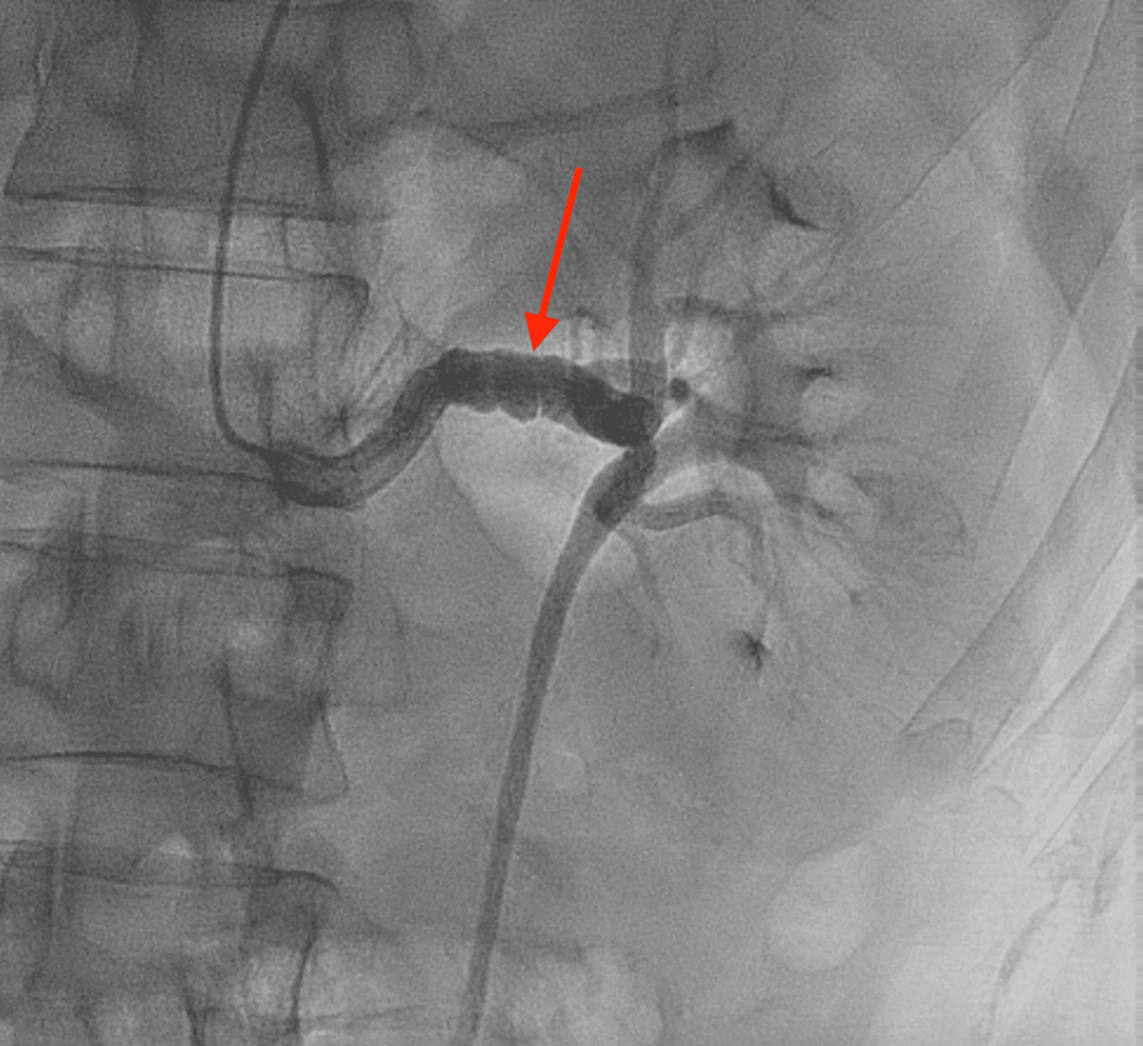
Renal artery angiogram in 2021 showing "string of bead” appearance of left renal artery characteristic of fibromuscular dysplasia (red arrow)

**Figure 2:**
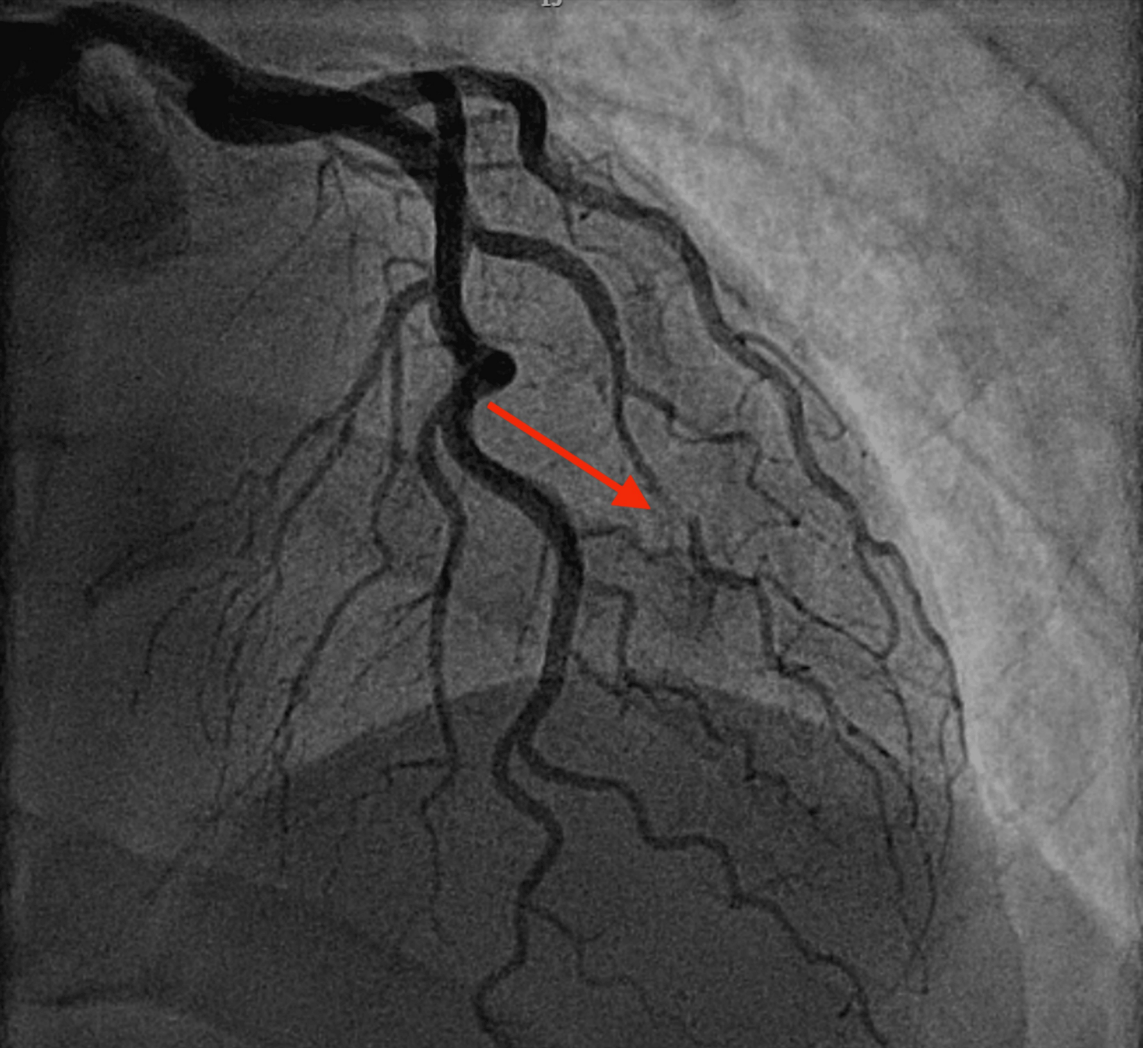
Coronary angiogram in 2021 demonstrating the first SCAD of the diagonal branch of the left anterior descending artery (red arrow) SCAD: spontaneous coronary artery dissection

**Figure 3:**
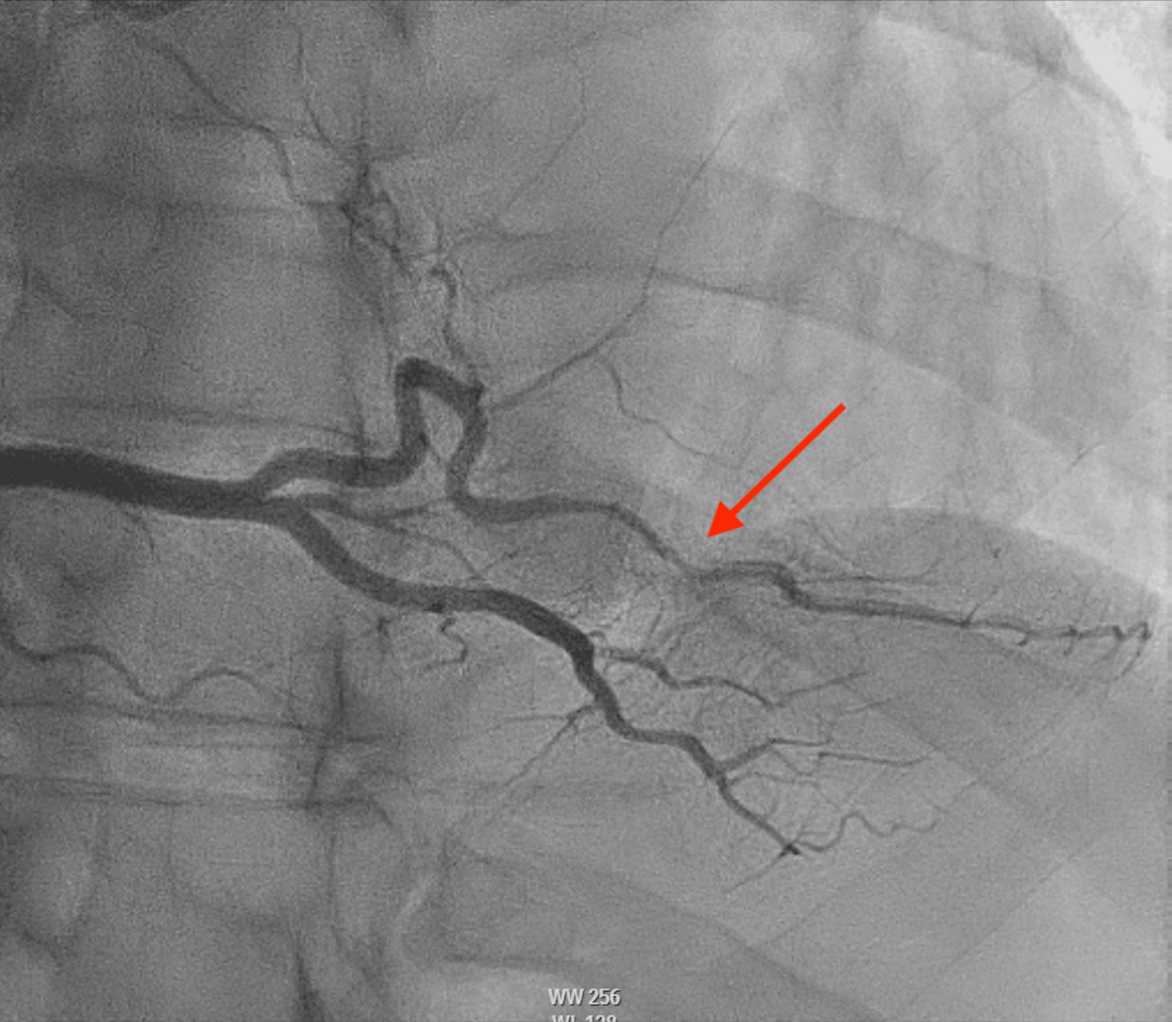
Coronary angiogram in 2022 demonstrating the second SCAD of the right posterolateral branch of the RCA (red arrow) SCAD: spontaneous coronary artery dissection; RCA: right coronary artery

**Figure 4:**
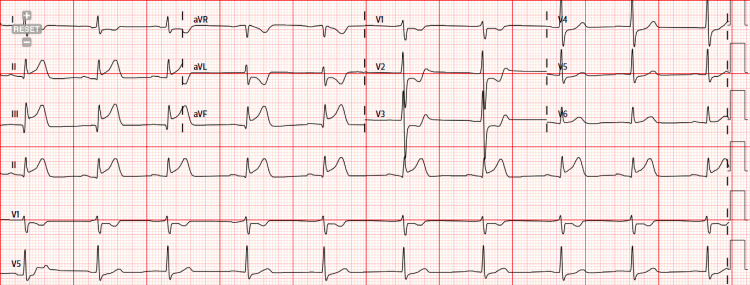
Electrocardiogram on initial presentation showing ST elevation myocardial infarction in the inferior leads

A 39-year-old male patient with a past medical history of FMD (Figure 1), SCAD of the diagonal branch (Figure 2), SCAD of the right posterolateral artery (Figure 3), left ophthalmic internal carotid artery aneurysm, and former tobacco and cocaine use was brought in by ambulance to the emergency department with severe non-radiating, mid-sternal chest pressure related to exertion. He described a sensation comparable to his prior episodes of SCAD in 2021 and 2022. En route to the hospital, he was given an aspirin load and was found to be hypotensive. The physical exam was unremarkable. Labs were notable for an initial troponin and B-type natriuretic peptide that were normal, but an electrocardiogram showed ST-elevations in the inferior leads consistent with an inferior ST-elevation myocardial infarction (Figure 4).

**Figure 5:**
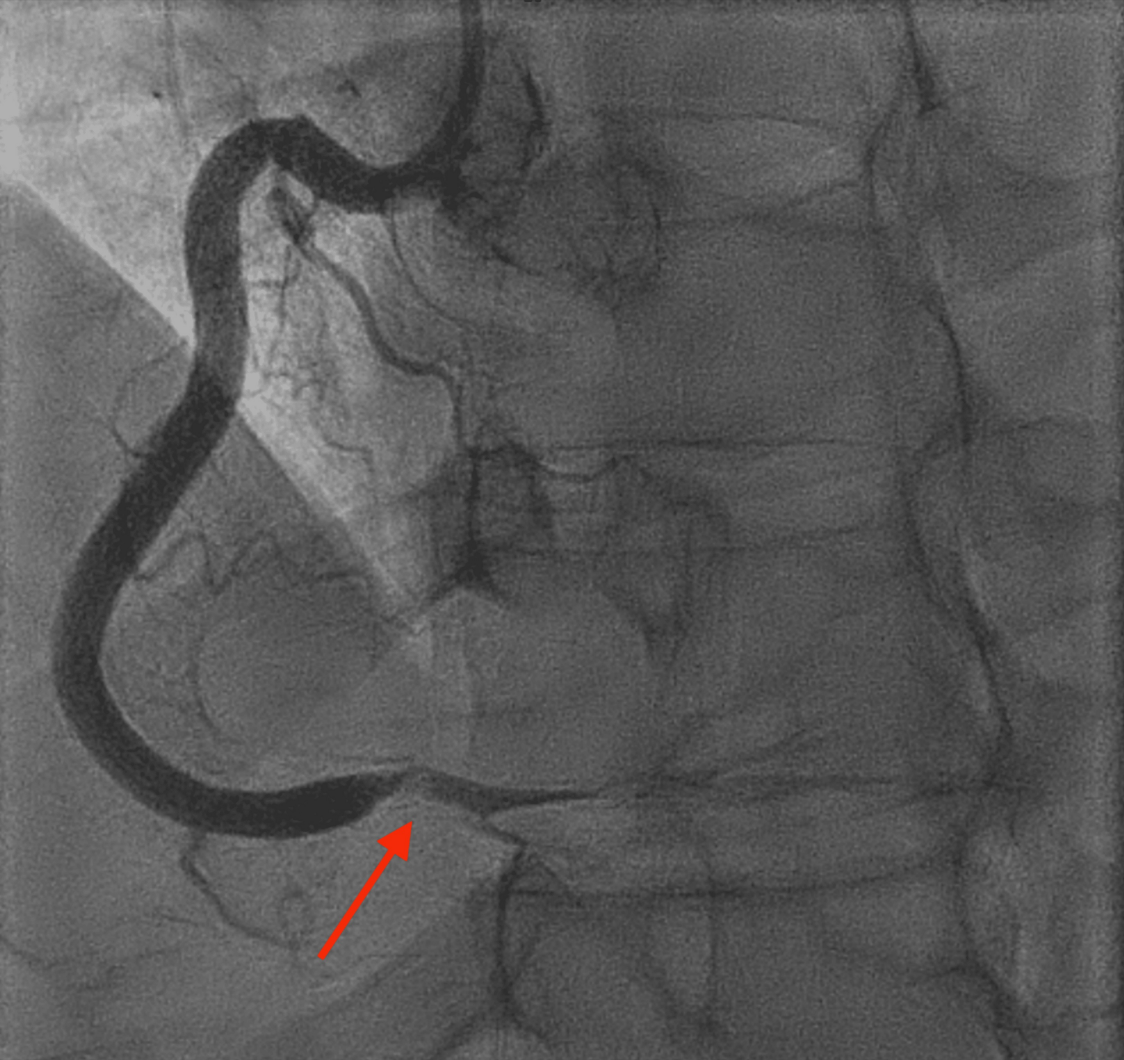
Coronary angiogram of current presentation demonstrating SCAD of the distal RCA (red arrow) SCAD: spontaneous coronary artery dissection; RCA: right coronary artery

Given concerns about ACS, the patient was started on a heparin drip (12 units/kg/hour). The patient was taken to the cardiac catheterization lab for an invasive coronary angiography that showed recurrent SCAD involving the distal right coronary artery (RCA) (Figure 5). Although the RCA was a large caliber vessel with TIMI (thrombolysis in myocardial infarction) 2 flow, the angiogram revealed an abrupt change in the distal RCA caliber with diffuse narrowing before the bifurcation of the right posterior descending artery and posterior left ventricular branches. Given the size mismatch and dissection extending further distally, the risks of a stent were deemed high, and he was treated medically with therapeutic intravenous heparin and aspirin, with symptoms resolving.

Electrocardiographic ST-segment changes returned to baseline, and troponin I peaked at 138 ng/mL and trended down. A transthoracic echocardiogram showed a mildly reduced left ventricular ejection fraction of 45% with mild global hypokinesis. The patient refused dual antiplatelet therapy and was discharged on aspirin and metoprolol. The patient had a repeat angiogram five months later which showed complete vessel healing with no residual dissection of the distal RCA. 

## Discussion

Diagnosing SCAD presents a challenge, and there may be a lower index of clinical suspicion of SCAD in men due to the reported low prevalence. A Canadian study evaluating 1,173 patients with SCAD found that men made up only 10.5% of the patients, while another study assessing the prevalence of SCAD in a database from catheterization labs found that men made up only 0.07% [4,5]. Additionally, the mean age of the male population affected by SCAD was 49.4 years, significantly younger than that of the women affected [4]. While SCAD is predominantly reported in women, it is essential to recognize that underdiagnosis may contribute to the lower incidence in men, as reflected by the variable incidence rates. It is important to have a high degree of suspicion for SCAD in young male patients presenting with acute coronary syndrome with no other significant cardiovascular risk factors. 

Some of the common risk factors in women with SCAD include oral contraceptives and emotional distress, while men who participate in intense isometric exercises and smoking are more likely to develop SCAD [6]. FMD can also predispose patients to SCAD by causing abnormal cellular growth in the arterial wall of coronary vessels, which makes the vessel more vulnerable and at risk of developing dissections [7]. There is a strong genetic link between SCAD and FMD as they both share the phosphatase and actin regulator 1 (*PHACTR1*) gene [7]. This gene influences endothelin-1 expression, which plays a role in vascular tone and remodeling, implying a shared pathophysiological mechanism in vascular remodeling and arterial dissection susceptibility between FMD and SCAD [7]. Although FMD predominantly affects women, men can present with FMD, which can involve different vascular beds and manifest into life-threatening aneurysms and dissections [8]. Men with FMD tend to be predisposed to more severe manifestations of SCAD and higher rates of recurrence due to higher levels of coronary artery tortuosity [8]. This can lead to life-threatening complications such as ventricular arrhythmias, cardiogenic shock, and sudden cardiac death. 

Recurrent SCAD is when there is another episode of SCAD after an initial dissection, which usually involves different coronary artery segments and occurs more than 30 days after the initial dissection [9]. The recurrence rates of SCAD vary, ranging from 8% to 27%, but these rates can be higher in patients with FMD [9]. There are no current universal guidelines for the management of SCAD. If the patient is hemodynamically stable, they should be managed conservatively with antiplatelet and beta-blocker therapy and inpatient monitoring due to the early risk of recurrent myocardial infarction [10]. Conservative management with antiplatelet therapy and beta blockers has been shown to be protective against recurrent SCAD due to its action on the heart and vasculature [11]. Beta-blockers can have beneficial effects by decreasing the contractility of the heart and intracoronary shear stress, which can theoretically reduce the degeneration of the aneurysm [11]. However, our patient had a past medical history of FMD and continued to develop recurrent SCAD despite being on conservative management with aspirin and beta-blockers. There is a need to study the pathophysiologic correlations of FMD and SCAD in men further and evaluate newer therapies for managing recurrent SCAD.

## Conclusions

SCAD in men is rare but could be underdiagnosed and should remain high in the differential in young patients presenting with chest pain without traditional cardiovascular risk factors. Timely diagnosis and appropriate management are critical to prevent recurrence, as it is common to develop multiple episodes of dissection in different coronary arteries in patients with FMD. While the management of recurrent SCAD currently involves conservative medical therapy, including antiplatelet agents and beta-blockers, there is a need to study the link between FMD and SCAD further and develop newer therapies to prevent recurrence. Close follow-up is essential to mitigate the risk of recurrence and adverse cardiac events.
